# Two Biosensors for the Determination of VEGF-R2 in Plasma by Array SPRi

**DOI:** 10.3390/molecules28010155

**Published:** 2022-12-24

**Authors:** Lukasz Oldak, Beata Zelazowska-Rutkowska, Anna Lesniewska, Piotr Mrozek, Marcin Skoczylas, Zenon Lukaszewski, Ewa Gorodkiewicz

**Affiliations:** 1 Faculty of Chemistry, Bioanalysis Laboratory, University of Bialystok, Ciolkowskiego 1K, 15-245 Bialystok, Poland; 2 Doctoral School of Exact and Natural Science, Faculty of Chemistry, University of Bialystok, Ciolkowskiego 1K, 15-245 Bialystok, Poland; 3 Department of Pediatric Laboratory Diagnostics, Medical University of Bialystok, Waszyngtona 17, 15-274 Bialystok, Poland; 4 Faculty of Mechanical Engineering, Bialystok University of Technology, Wiejska 45C, 15-351 Bialystok, Poland; 5 Faculty of Computer Science, Bialystok University of Technology, Wiejska 45A, 15-351 Bialystok, Poland; 6 Faculty of Chemical Technology, Poznan University of Technology, pl. Sklodowskiej-Curie 5, 60-965 Poznan, Poland

**Keywords:** array SPRi biosensor, VEGF-R2, angiogenesis marker, blood plasma, silver/gold chip

## Abstract

Vascular endothelial growth factor receptor 2 (VEGF-R2) is a marker of angiogenesis and metastasis of cancer. Two biosensors for the determination of VEGF-R2 in plasma have been developed. One of them is based on a pure gold chip, and the other on a silver/gold bimetallic chip; both have the receptor, monoclonal rabbit antibody specific for human VEGF-R2, attached to the chip via a cysteamine linker. The biosensor with the gold chip exhibits linearity of the analytical signal between 0.03 and 2 ng/mL, a precision of 1.4% and recovery between 99% and 102%. The biosensor with the bimetallic chip exhibits linearity between 0.03 and 1 ng/mL, a precision of 2.2% and recovery between 99% and 103%. Both biosensors tolerate a 1:100 excess of VEGF, VEGF-R1 and VEGF-R3. Both biosensors were validated by parallel determination of VEGF-R2 in 27 different plasma samples using the ELISA immunosensor assay, with very good agreement of the results. Thermodynamic parameters of the interaction of VEGF-R2 with the antibody were determined by QCM (Quartz Crystal Microbalance) and SPRi (Surface Plasmon Resonance imaging) measurements.

## 1. Introduction

Vascular endothelial growth factor receptor 2 (VEGF-R2), together with VEGF-A, plays an important role in physiological and pathological angiogenesis, including tumor angiogenesis [1] that is, in the formation of new blood vessels. A growing tumor requires an enhanced stream of nutrition. Therefore, it forms a network of new blood vessels, using VEGF-R2 for this purpose. Increased VEGF-R2 concentration is a symptom of the occurrence of metastasis, the most dangerous stage of cancer. Therefore, one aim in the treatment of cancer patients is the lowering of VEGF-R2 concentration by the introduction of VEGF-R2 inhibitors [1]. VEGF-R2 is a transmembrane receptor consisting of the extracellular ligand-binding domain, a transmembrane domain, a tyrosine kinase domain [2] and 1356 amino acids (200 kDa). In spite of being fixed in the membrane, the VEGF-R2 receptor is present in the plasma of patients with pancreatic cancer [3] and in the serum of patients with endometrial cancer [4]. Furthermore, changes in serum levels of VEGF-A, VEGF-R1 and VEGF-R2 were studied in pediatric acute lymphoblastic leukemia using ELISA. The role and concentrations of VEGF-R2 in this tumor have not yet been fully described. The studies were conducted on samples from patients at the time of diagnosis (day 0) and at the end of the induction phase (day 35), and in the control group. The median VEGF-R2 concentration on day 0 was 17,577.5 pg/mL, and on day 35 it was 20,507.5 pg/mL, while in the control group the median VEGF-R2 concentration was 22,267.5 pg/mL [5]. A study was also conducted to determine the effect of VEGF-R2 on ovarian cancer-free survival and recurrence, using immunohistochemistry. Based on the results of the study, the authors concluded that the VEGF-R2 status was associated primarily with the type of tumor and recurrence of the disease. It also appears that positive expression of VEGF-R2 results in positive progression-free survival [6]. In addition, a Western Blot study showed higher VEGFR-2 concentrations in ovarian cancer tumors at FIGO stages I and II compared to FIGO stages III and IV [7]. Available data show that VEGF-R2 concentration in serum ranges from 6 to approx. 23 ng/mL [4,5]. To our knowledge, quantitative VEGF-R2 determination is a poorly researched area. To date, an electrochemical sensor capable of detecting VEGF-R2 has been developed [8], which enables the determination of VEGF-R2 within the range of 0.4–100 pM (0.8–20 ng/mL). The ELISA immunoassay has also been used for this purpose [3,4]. The development of new analytical tools for VEGF-R2 determination can be expected to facilitate wider application of this promising biomarker.

An antibody or antigen may be immobilized on the surface of the biosensor. Due to the nature of this research, we will focus on the binding of antibodies to the surface of the biosensor. In Table 1, we present known methods of binding antibodies to the biosensor surface, along with their advantages and disadvantages.

Array SPRi is a technique used to determine molecular biomarkers in body fluids, in what is called ‘liquid biopsy’. The technique is gradually gaining importance in clinical investigations, e.g., [10,11,12,13]. Almost 30 biosensors have been developed for use with array SPRi or conventional SPR, including sensors for the determination of the known cancer biomarkers CA-125 [14], HE-4 [15] and CEA [16], as well as new promising cancer biomarkers such as circulating microRNA [17] or exosomes [18] in the case of breast cancer biomarkers. The array SPRi technique enables the determination of biomarkers within the ranges of concentration characteristic for cancer patients and for healthy subjects, without the need for any biomarker accumulation or signal enhancement (e.g., with gold nanoparticles). The technique differs from classic fluidic SPR in two respects: (i) the biosensor is formed ex situ, while in classic SPR it is formed in situ during measurement; (ii) the SPRi measurement is performed after the removal of processing liquids (in classic SPR measurement it is performed in the presence of processing liquids). By using an array of measuring points, several samples (usually nine) can be measured simultaneously. An advantage of the technique is the simple construction of the biosensor. Thus, array SPRi is potentially a suitable tool for the determination of VEGF-R2, provided that a suitable biosensor can be developed.

Literature reports show that the use of a bimetallic biosensor should primarily increase the sensitivity of the method based on it. As in examples of such studies, we cite a biosensor based on bimetallic Pd@Au rods for the determination of pesticides [19], optimization of the thickness of the Ag/Au bimetallic layer in order to achieve the highest possible sensitivity [20], and increasing the sensitivity of a biosensor based on nanocomposites of titanium, graphene and barium using a bimetallic configuration (Ag/Au) [21].

The aim of this work was to develop a new method for the quantitative determination of the circulating factor VEGF-R2 based on the use of SPRi biosensors and determination of its analytical parameters. It is one of the few methods of VEGF-R2 determination in natural samples. The successful development of a tool for the determination of circulating VEGF-R2 in plasma/serum should facilitate the detection of cancer metastasis. The medical industry have the right to expect the creation of new tools for cancer detection and grading from the analytical chemistry. To increase the chances of success, two versions of the biosensor were investigated: one built on a standard commercially available gold chip and the other using a bimetallic chip with gold and silver in the correct proportions. The silver–gold chip is just as suitable as the pure gold chips for building biosensors for use with the SPRi array. Due to the difference in plasmonic properties, these two chips created different calibration conditions, which made it possible to increase the sensitivity of the newly developed method. The work presents, for the first time, thermodynamic studies of the biological system (ligand-VEGF-R2) with the use of QCM and SPRi as well as the characteristics of the sensor surface.

## 2. Results

### 2.1. Sensors Preparation

**Figure 1:**
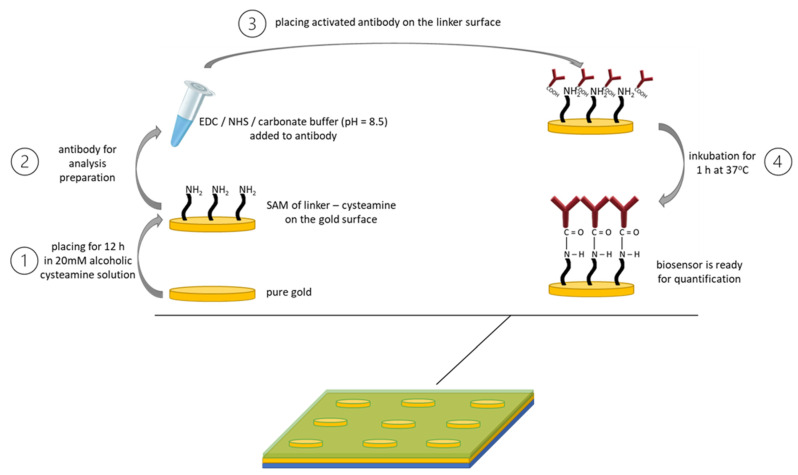
Scheme and conditions of linker (cysteamine) and ligand (monoclonal antibody) immobilization.

Gold and bimetallic chips were used in the research. Chips with pure gold as the plasmonic material were purchased directly from the manufacturer (SSens, http://www.ssens.nl/, accessed on 10 March 2021). The bimetallic chips consisted of a glass base (microscope slides, n_D_ = 1.51), a Cr adhesive layer (1 nm), an Ag layer (40 nm) and an Au layer (4 6 nm). On each of the chips, special separating layers were printed to create nine independent measurement locations. Elpemer SD 2457 polymer was used for this purpose. The exact procedure for preparing the sensors has been described in previous articles [22,23]. The immobilization of the linker, cysteamine, and the ligand–monoclonal antibody, for both sensors, was carried out according to Figure 1.

The first step was to coat the sensor with a self-assembled monolayer of the linker, in our case cysteamine (1). Then, the EDC and NHS solutions were mixed in a 1:1 volume ratio in the presence of a carbonate buffer to ensure the appropriate pH of the reaction medium, and then the mixture was introduced into the antibody solution, after which the whole product was mixed again (2). The third step was to place the previously prepared mixture onto the sensor with a linker layer. It was incubated for 1 h at 37 °C (3). After completion of the above steps, the biosensor was ready to be used for quantitative determinations in biological fluids (4).

The components of the SPRi spectrometer are a light source (diode laser, λ = 635 nm) and a system of lenses focusing the incident radiation, and polarizers, which are responsible for extracting the polarization of p or s radiation. Then, the radiation with the appropriate polarization is directed to an equilateral glass prism made of BK-glass 7, on which the biosensor is placed after prior application of an immersion oil with a refractive index of nD = 1.54 (consistent with the refractive index of the prism). The immersion oil prevents the formation of a glass–air interface between the prism surface and the base of the biosensor. The radiation reflected from the surface of the biosensor goes to the detector, which is a monochrome CCD camera with a resolution of 1.4 MP. ImageJ 1.51k (NIH Image) software is used to process the images in order to obtain an analytical signal.

The SPR curves were recorded by experimentally forming successive layers of the biosensor components on one of its active sites and analyzing the resulting images. The data obtained were used to plot the SPR curves using WinSpall software.

The formation of subsequent layers of the biosensor is evidenced by shifts of the SPR curves characterizing a given layer towards higher angle values, compared with the SPR curve for the biosensor layer immediately preceding the one currently being analyzed.

**Figure 2:**
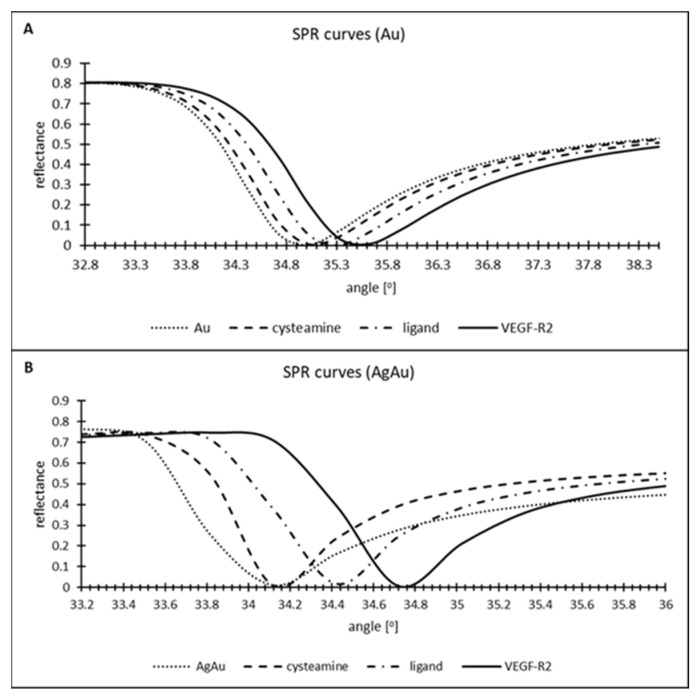
SPR curves plotted with WinSpall. (**A**) gold chip; (**B**) bimetallic chip.

Figure 2 shows the SPR curves for both biosensors. In the case of the biosensor with only a gold layer as the plasmonic material, the shifts of the SPR angle between consecutive individual sensor elements are of the order of 0.1–0.2°. In the case of the biosensor with silver and gold, an imperceptible difference in the SPR angle change is observed between the metal layer and cysteamine. The successive layers of the biosensor cause shifts of 0.3°. Moreover, the minima of the SPR curves in this case are sharper. All of these properties suggest that the use of two plasmonic metals leads to an increase in the sensitivity of the analytical method, and therefore to more accurate results.

Table 2 shows the values of the parameters used to model the SPR curves (Figure 2) in order to fit them to the experimental data. The table contains information on the thickness of individual layers of the biosensor, and values of the real and imaginary parts of the permittivity.

Taking into account the characteristics of the surface, the determined thicknesses of individual layers should be treated as averaged values. Previous studies have shown that gold and bi-metallic chips differ in their roughness. The RMS roughness values were as follows: 2.12 nm for the bimetallic chip and 0.15 nm for the gold chip [23]. This may have a potential impact on the attachment of individual molecules to the biosensor surface and the availability of antibody molecules for the analyte. A less rough surface ensures a more even distribution of particles on the surface of the biosensor. They are densely packed and steric hindrance can occur; hence not all ligand molecules have the opportunity to bind to the analyte. On the surface with higher roughness, elevations and depressions are formed. They provide better separation of molecules binding to the biosensor surface, which results in more ligand molecules having a chance to bind to the biosensor surface and thus more analyte molecules having a chance to interact with the ligand. Steric hindrance is also minimized. However, such a surface has the disadvantage of possible non-uniformity. The point here is that the surface of a bimetallic chip may contain islands composed of Ag/Au and patches where there is only silver on the surface, or there is so little gold that it is impossible to attach a thiol to the surface of the biosensor. Such a phenome-non is unlikely to be observed on uniform surfaces. Failure to attach a thiol will result in the lack of a ligand in a given place and further inability to capture the analyte from the solution. Therefore, in order for the advantages of a bimetallic chip to be fully exploited, it is necessary to carry out detailed and comprehensive control of its surface during production.

To prevent non-specific adsorption on the biosensor surface, which may negatively affect the results (e.g., false positives), 3 µL BSA (1 mg/mL) was applied to the active sites of the biosensor before the ligand–analyte binding step, for about 10 min. Next, the surface of the active sites was rinsed with water to remove excess BSA.

### 2.2. Saturation of Sensor Surfaces with Antibody (Ligand)

**Figure 3:**
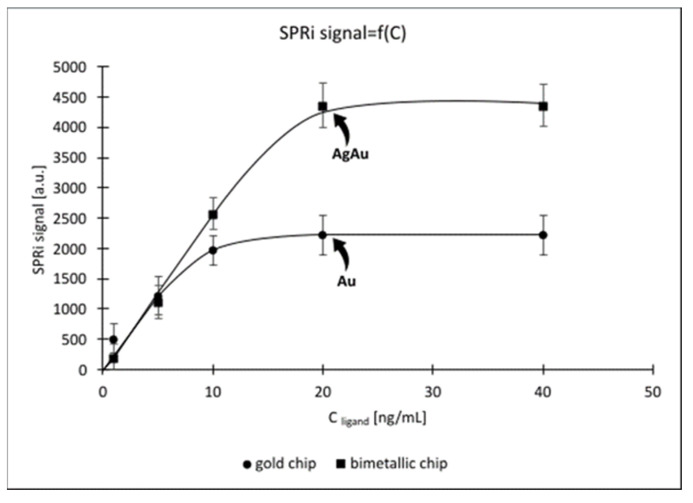
Saturation of the sensor surface with ligand. VEGFR-2 concentration was 5 ng/mL, pH = 7.40.

The curves were obtained in a neutral environment (physiological pH = 7.4). Eight standard ligand solutions were prepared, which were then placed on a previously prepared sensor with a cysteamine layer as the linker. The whole resulting product was incubated for one hour at 37 °C. After this time, excess ligands were removed by washing the biosensor surface with milliQ water and HBS-ES solution. An equal concentration (C = 5 ng/mL) of VEGF-R2 was applied to the active sites of the biosensor. The concentration of the analyte was lower than the maximum concentration of the surface saturation with the ligand due to the fact that the method of immobilization of the ligand to the surface (random immobilization) does not guarantee that all molecules will be bound to it. Therefore, in order not to obtain an artificially high signal caused by non-specific adsorption, the concentration of the analyte was lower than the concentration of the ligand. If it were too low, the saturation curve would not reach a plateau. This would require repeating the experiment with a correspondingly higher concentration of the analyte. There was also a reference site, to which blank (PBS) was applied. The time allowed for interaction between the ligand and VEGF-R2 was 10 min, after which the biosensor surface was washed again with milliQ water and HBS-ES solution. Figure 3 shows the ligand saturation curves of the sensor surface.

Both sensors lead to a characteristic monomolecular adsorption curve (as described by Langmuir). This suggests the formation of a ligand monolayer, which is desirable due to the characteristics of the tests. The plateau is established at a ligand concentration of 20 ng/mL for both biosensors. Above this concentration, further binding of VEGF-R2 to the ligand is no longer possible, since it is impossible to immobilize more ligands and all available ligands on the surface of the biosensors have been bound.

The maximum detector response of the instrument occurs only when all ligand binding sites are occupied by the analyte. It depends on the number of ligand particles that have been immobilized on the sensor surface, as well as the mass of ligand and analyte, i.e., the size ratio of the ligand–analyte complex. Additionally, the detector response depends on the number of ligand binding sites. There is also a risk that binding of the analyte to a ligand-dense surface may result in the screening of more than one ligand binding site (e.g., the formation of random protein agglomerates). In such a situation, the maximum detector response signal calculated from equation (1) will be lower than that obtained in the course of the experiment.

(1) $$SPRi_{max}=\frac{SPRi_{ligand}M_{analyte}Valency_{ligand}}{M_{ligand}}$$

where:

SPRi_ligand_—maximum SPRi signal for maximum ligand coverage of the sensor surface; [Au: 2040.20 au; AgAu: 3254.05 au]—the response of the device detector when there is only a linker and a ligand on the surface of the biosensor;

M_analyte_—molar mass of the tested analyte [200 kDa];

Valency_ligand_—the number of ligand binding sites [1];

M_ligand_—molar mass of the ligand [151 kDa];

SPRi_max_ is, respectively, for the gold chip SPRi_max_ = 2702.24 au, and for the bimetallic chip SPRi_max_ = 4310 au.—the response of the device’s detector when a ligand–analyte complex is formed on the surface of the biosensor.

In general, the amount of ligand actually active is unknown, and is highly variable depending on the immobilization technique used. Covalent coupling chemistry gives the best results in terms of obtaining highly active biological surfaces. A required condition, however, is that the reactive group that forms the covalent bond be as far away as possible from the group that interacts with the analyte [24,25,26].

The maximum SPRi signal (SPRi_max_) that the device detector can give for the tested systems, assuming that the ligand is covered with a monolayer of analyte, was calculated from the following formula (1). The SPRi_max_ values were used when constructing the saturation plots and the calibration relationships for both biosensors.

The SPRi_max_ value indicates the maximum signal that the device should receive with the assumed measurement parameters.

Since the theoretically determined SPRi_max_ values are in agreement with the experimental values (Figure 3), we can conclude that the ligand (antibody) used has one binding site. This assumption is supported by the fact that the value of the maximum SPRi signal obtained as a result of the experiment (2683.70 Au; 4302.42 Ag/Au) is consistent with the value calculated theoretically (2702.24 Au; 4310.00 Ag/Au). If the antibody had two binding sites, each of the experimental signals would be doubled

### 2.3. Method Calibration

**Figure 4:**
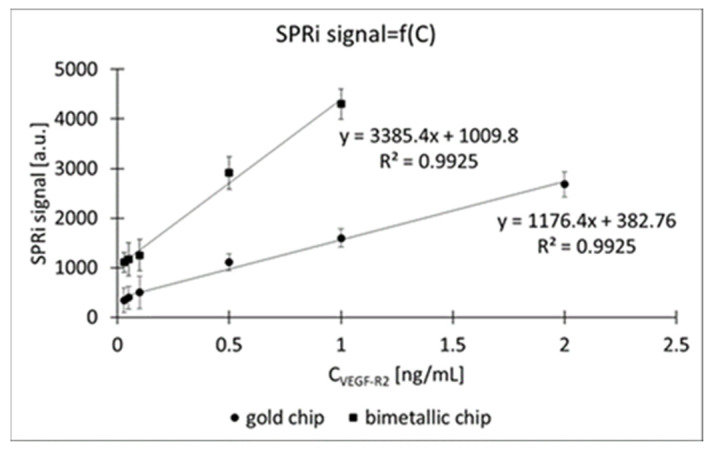
Working range of the calibration curves. Ligand concentration was 20 ng/mL, pH = 7.40.

**Figure A1:**
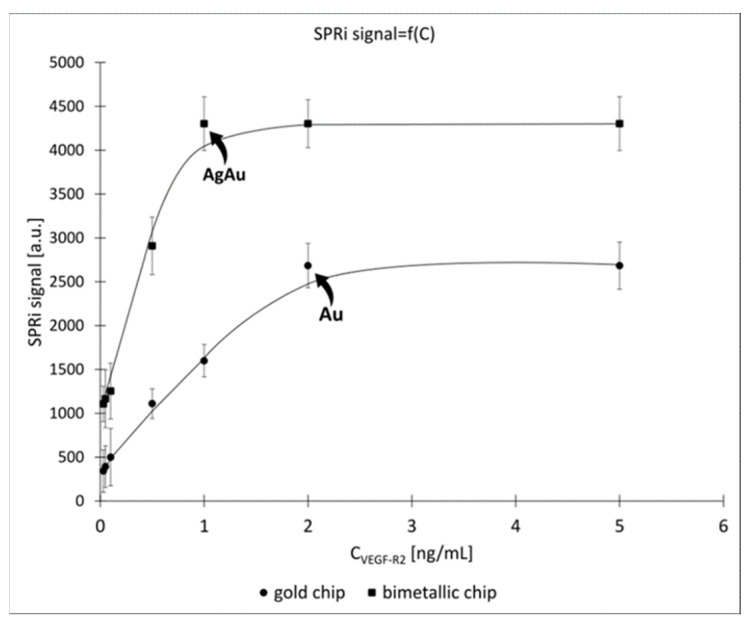
Calibration curve over the full concentration range. Ligand concentration was 20 ng/mL, pH = 7.40.

Eight standard solutions of VEGF-R2 with concentrations of 0.03, 0.05, 0.10, 0.50, 1.00, 2.00 and 5.00 ng/mL were prepared, and these were applied to the individual active sites of the biosensor. One of the sites was used as a reference (with PBS applied). The working range of the calibration curves is shown in Figure 4, while the full range of the calibration curves is shown in Figure A1 in the Appendix A. The plateaus were determined at the following concentrations of VEGF-R2: for the biosensor with only gold as the plasmonic material, C_VEGF-R2_ = 2 ng/mL; and for the biosensor coated with silver and gold, C_VEGF-R2_ = 1 ng/mL.

From the above calibration relationship, the working range was selected (from LOQ to 2 ng/mL for the gold chip, and from LOQ to 1 ng/mL for the bimetallic chip), and regression equations were determined and used for further analyses.

The calibration curve characterizing the silver- and gold-coated chip has a slope approximately 2.9 times greater than that of the calibration curve of the gold chip (3385.4 vs. 1176.4). This is another observation suggesting that the combination of two plasmonic metals increases the functional value of the constructed biosensor. The functional value of the biosensor is increased due to the increase in sensitivity, which is observed when two plasmonic metals are used. Silver is considered one of the best plasmonic metals with the lowest ohmic losses. Its SPR curve looks steeper and has a more accurate minimum compared with a 100% gold plate [27]. However, it does not have the ability to attach thiols to its surface (as for example, in our case, cysteamine) and is quickly oxidized. Therefore, in our research, a thin layer of gold with a thickness of 6 nm sputtered onto the silver layer. This layer had a double function: to enable binding of the thiol to the surface of the biosensor and to protect the silver surface against oxidation. By using such a combination, we exploit the advantages of two plasmonic metals. Silver makes it possible to achieve maximum differences in the analytical signal, in relation to the difference in analyte concentrations, while gold allows the thiol to be attached to the surface of the biosensor and protects silver against oxidation.

### 2.4. Methods Precision, LOB, LOD, LOQ

The precision of the developed methods was determined by applying standard solutions with appropriate concentrations of the reference material (C_RM_) to the active sites of the biosensors. The concentrations used corresponded to the endpoints of the calibration curves and the midpoints. For each sample, 10 independent measurements were made, the mean concentration value (C_quant_), standard deviation (SD) and relative standard deviation (RSD) were calculated, and the recovery value (REC) and the coefficient of variation (CV) were determined. A further essential validation step is the determination of the limit of blank (LOB), limit of detection (LOD) and limit of quantification (LOQ). The values are summarized in Table 3.

The very good precision of the developed methods is demonstrated by the REC values, which are in the range 100.00–112.00%. The CV was used to compare the volatility. The lowest variation was found at the 2.000 ng/mL point for the gold chip (CV = 0.30%), and the highest at 0.050 ng/mL for the bimetallic chip (CV = 10.70%).

The limit of blank (LOB) was determined on the basis of 10 measurements with a zero concentration of the analyte tested (pure PBS). The LOB value indicates the permanent systematic error. LOB was determined using the formula:

*LOB* = *AVERAGE*
*BLANC* + 1.645 ∙ *SD* (2)

LOD was determined by measuring 10 samples of PBS supplemented with the lowest concentration of VEGF-R2 that could be captured by the detector (C = 0.005 ng/mL). The arithmetic mean of the obtained results was calculated, and the SD was determined. The LOD was calculated from the following relationship:

*LOD* = 0 + 3 ∙ *SD* (3)

LOQ was determined using the equation:

*LOQ* = 3 ∙ *LOD* (4)

The LOB, LOD and LOQ values for both biosensors are the same, which indicates that in the lowest concentration range, the type of plasmonic metal does not play a significant role.

### 2.5. Recovery by the Method of Standard Addition

The first stage of this validation step was the quantification of VEGF-R2 in a randomly selected control sample (C_control_) consisting of plasma taken from smokers (diluted two times). Then a threefold excess of VEGF-R2 (C_add_) was added, and five independent measurements of the concentrations of the spiked samples were made. The recovery value (REC) and standard deviation (SD) were then calculated. The results are presented in Table 4.

(5) $$REC=\frac{C_{quant}-C_{control}}{C_{add}}\times 100\%$$

Small standard deviations indicate the high precision of the developed methods, while the good agreement between the experimentally determined concentrations and the theoretical concentrations proves the methods’ accuracy.

### 2.6. Selectivity

**Figure A2:**
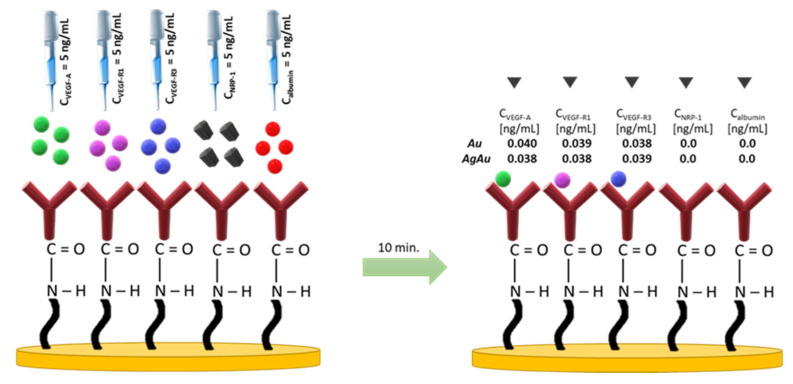
Schematic diagram of interaction of the interferent with the antibody. Ligand concentration was 20 ng/mL, pH = 7.40.

The selectivity of the developed methods was tested for both chips (gold and bimetallic: Ag/Au). The first step was to test the selectivity of the antibody used against individual components of the VEGF family. Potential interferents—VEGF-A, VEGF-R1 and VEGF-R3—were placed on the chip with the antibody. The procedure was also repeated for NRP-1 and human albumin. The interferent concentrations were 5 ng/mL. The experiment is presented schematically in Figure A2 in the Appendix A.

The obtained concentration values after the antibody–antigen interaction are not much higher than the LOQ (0.03 ng/mL) of the developed methods for the VEGF-A protein and other VEGF-R receptors. Therefore, it can be concluded that these do not have a major impact on the quantification of VEGF-R2 in body fluids. The remaining interferents tested did not react with the antibody in any way.

**Figure 5:**
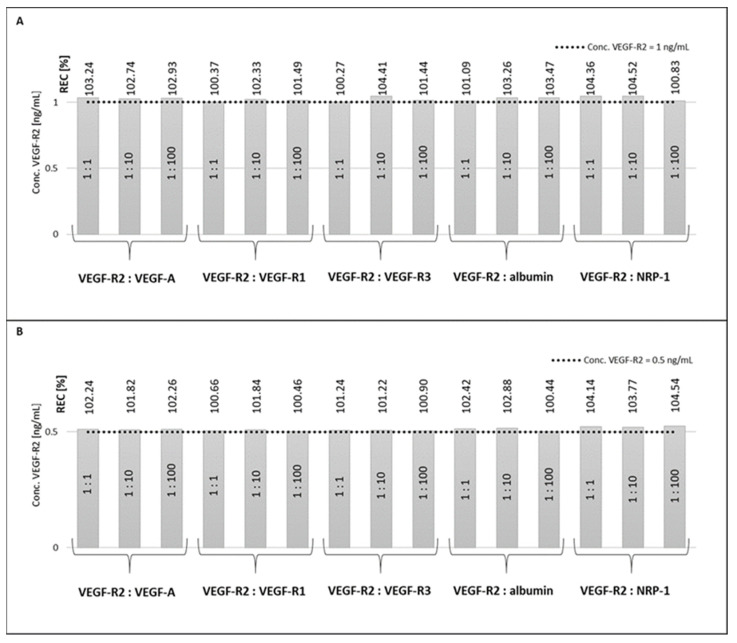
Selectivity of the developed methods, [VEGF-R2: interferent]. (**A**) gold chip; (**B**) bimetallic chip.

Next, a series of [VEGF-R2: interferent] solutions were prepared with molar ratios of concentrations [1:1], [1:10] and [1:100], where the concentrations were always selected so that the expected VEGF-R2 concentration was 1 ng/mL in the case of the gold chip, and 0.5 ng/mL in the case of the bimetallic chip, which is marked with a dashed line in the figure. Recovery values (REC) were calculated. The results of the tests are presented in Figure 5.

The REC values for both methods are in the range 100–105%. Therefore, we conclude that no excess of interferent interferes with the correct, selective operation of the biosensor.

**Figure A3:**
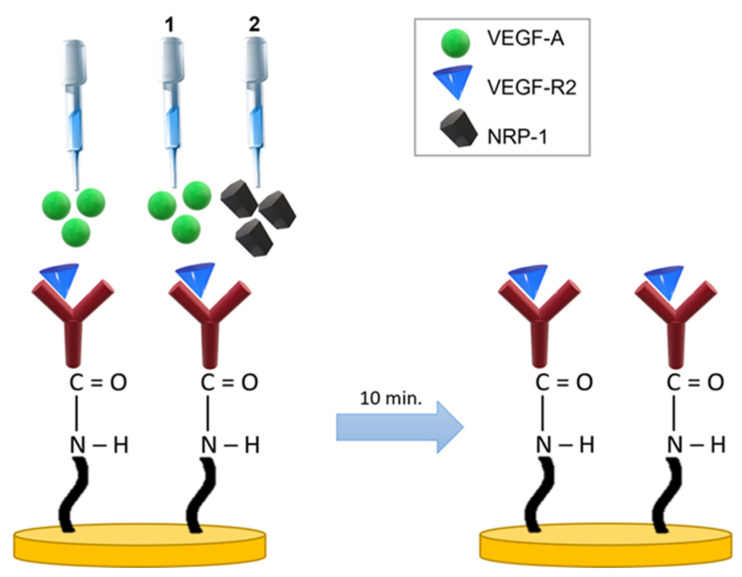
Schematic diagram of testing of potential reactions of interferents with ligand-bound VEGF-R2. Ligand concentration was 20 ng/mL, pH = 7.40.

The next step in the research was to test whether VEGF-R2 already bound to the ligand (antibody) could react with potential interferents (VEGF-A and NRP-1). For this purpose, VEGF-A solutions were applied in various concentration ratios with respect to the determined VEGF-R2 concentration. The same was done for NRP-1. The first step was to apply the VEGF-A, followed by the NRP-1. The methodology is presented schematically in Figure A3 in the Appendix A.

**Figure 6:**
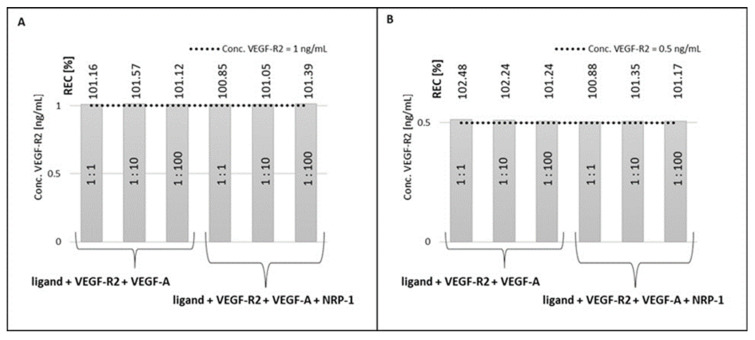
Selectivity of the developed methods, potential interferent reactions with ligand bound VEGF-R2. (**A**) gold chip; (**B**) bimetallic chip.

Figure 6 shows the results of the tests. The REC values of 100–103% indicate that neither VEGF-A nor NRP-1 react with ligand-bound VEGF-R2.

### 2.7. Thermodynamic Studies of the Biological System with the Use of QCM and SPRi and the Characteristics of the Sensor Surface

**Figure 7:**
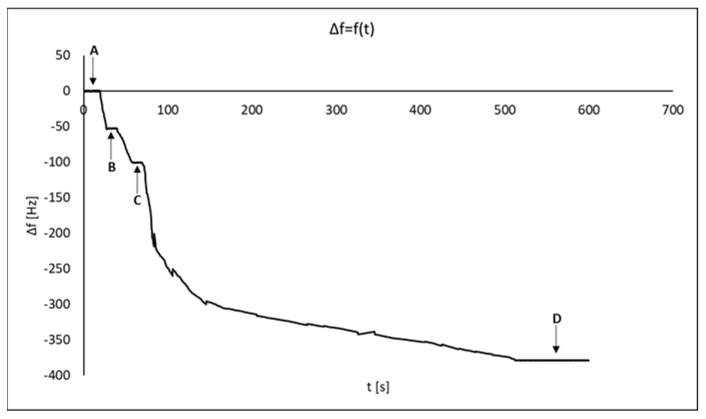
Graph of the dependence of frequency changes on time and successive layers of the biosensor. (A) pure gold; (B) cysteamine; (C) ligand; (D) VEGF-R2.

A quartz crystal microbalance (QCM) was used to determine the dissociation equilibrium constant (K_D_) and the association equilibrium constant (K_A_) of the ligand–VEGF-R2 complex. Figure 7 also provided information about the formation of successive layers of the biosensor.

Below, the thermodynamic parameters of the tested biological system will be summarized and compared, based on the use of QCM and SPRi (Table 5).

When conducting thermodynamic studies using SPR, care should be taken to ensure that the density of the sensor surface with the ligand capturing the analyte of interest is as low as possible, to avoid factors such as mass transfer or spherical blockage. Thermodynamic studies with the use of SPRi were carried out only for the gold chip. Based on the previously prepared surface saturation curve (Figure 3), the value SPRi_max_ was determined high enough for the detector of the device to be able to give reliable results, and low enough to obtain relatively loosely deposited ligands on the biosensor surface (SPRi_max_ = 1000 au). This ensures that we create a ligand layer with the lowest possible density on the biosensor surface, while obtaining a detector response that is not disturbed by factors that may falsify the measurements, e.g., mass transfer effects or steric hindrances. Then, we calculated what SPRi signal for the bound ligand (SPRi_ligand_) we expect from the detector, according to Equation (6):

(6) $$SPRi_{ligand}=\frac{SPRi_{max}M_{ligand}}{M_{analyte}Valency_{ligand}}$$

**Figure 8:**
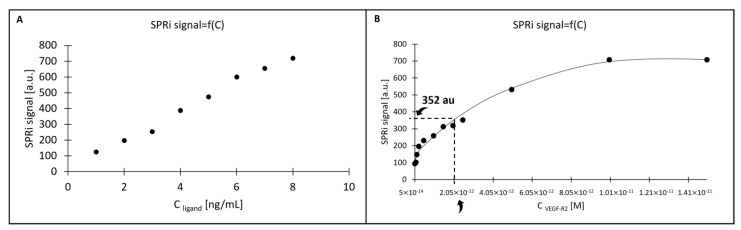
Curves for the determination of thermodynamic parameters by the SPRi method. (**A**) the detector’s response to the introduced ligand at various concentrations; (**B**) determination of the K_D_ value by SPRi.

The value SPRi_ligand_ = 755 au was obtained. A curve was plotted for the detector’s response to the introduced ligand at various concentrations (ranging from 1 to 8 ng/mL) (Figure 8A). As the SPRi signal should not exceed 755 au, 8 ng/mL was chosen as the optimal ligand concentration for the study. The next step was the application of the tested analyte in concentrations between 0.01 and 3.00 ng/mL, and determination of the K_D_ value, i.e., the analyte concentration which gives a signal corresponding to the saturation of no more than 50% of the available ligand. In our case, the relevant SPRi signal value was 352 au. (Figure 8B).

The point 0.5 ng/mL (2.05 × 10^−12^ M) was closest to the assumed value of the SPRi signal corresponding to the K_D_ of the biological system, causing the detector to respond with an SPRi signal equal to 352 au.

The value K_A_ of the system was determined from Equation (7):

(7) $$K_{A}=\frac{1}{K_{D}}$$

The results obtained for the thermodynamic characteristics of the studied ligand–VEGF-R2 system, obtained by means of QCM and SPRi, are summarized in the table below (Table 5). The table also includes information about the surfaces of the biosensors obtained by SPRi: the amount of ligand deposited on the surface (ligand_sites_) and the amount of functional ligand (ligand_functional_). These considerations are, of course, purely theoretical and assume the availability of all deposited ligands.

(8) $$*\;ligand_{sites}=\frac{SPRi_{ligand}}{M_{ligand}Valency_{ligand}}$$

(9) $$**\;ligand_{functional}=\frac{SPRi_{max}M_{ligand}}{SPRi_{ligand}M_{analit}}\times 100\%$$

The affinity of the recombinant NTV1 nanobody for domain 3 of VEGF-R2 was tested. The research led to a value of 49 ± 1.8 nmol/L (4.9 × 10^−8^ mol/L) [28].

The research methodology described in this article assumes random immobilization. Although theoretical considerations show that almost 100% of all ligands are functional, it should be remembered that SPRi is sensitive to mass changes on the surface of the biosensor. Thus, even when the antibody or test protein is completely or partially destroyed, it may retain the ability to bind to the surface of the biosensor or bind to its surface by physical adsorption. In this case, we will also get results indicating the activity of all ligands, but this will not be accurate. Thus, the result obtained should be regarded as an estimate only.

### 2.8. Determinations in Natural Samples

To verify the correctness of the developed methods of quantitative determination of VEGF-R2 in plasma, determinations were made with the use of a commercial ELISA test (Abcam, ab100665). The biosensor-based method required appropriate dilutions. For the gold biosensor, all samples were diluted two times, while in the case of the bimetallic biosensor, the samples were diluted four times.

To compare the concentrations obtained using the developed methods and those obtained with the use of a commercial ELISA test, scatterplots were produced, and the Spearman’s rank correlation coefficient (ρS) was calculated, with the statistical significance level of *p* < 0.05.

**Figure 9:**
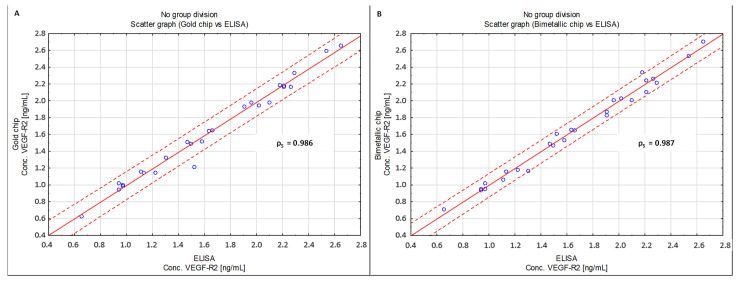
Scatterplot comparing the developed methods with ELISA and showing Spearman’s rank correlation coefficient ρS. (**A**) gold chip vs. ELISA; (**B**) bimetallic chip vs. ELISA.

**Figure 10:**
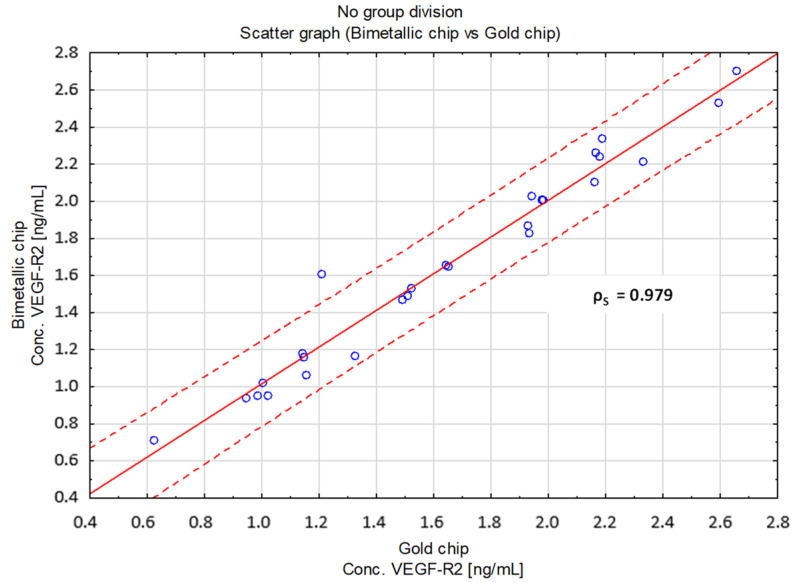
Scatter plot comparing both developed methods and showing Spearman’s rank correlation coefficient ρS.

Figure 9 shows a comparison of both newly developed methods for a commercial ELISA test, while Figure 10 shows a comparison of the VEGF-R2 determination method based on a gold and bimetallic biosensor.

In each of the analyzed cases, ρS is close to 1, which indicates very good agreement between the compared methods.

## 3. Discussion and Conclusions

Two biosensors were constructed as promising tools for the quantification of VEGF-R2 in plasma. Two metallic bases were used for the biosensors. The first was constructed on a commercial gold chip. In the second case, two plasmonic metals—silver and gold—were used, sputtered on a glass plate in accordance with the method described in a previous publication. The use of two plasmonic materials is beneficial mainly for increasing the sensitivity of the method. We observed this on the basis of the minima of the SPR curves, which were better spaced in the case of the bimetallic chip, than in the case where pure gold was used (Figure 2). Similarly, the calibration curve of the bimetallic chip is characterized by a sensitivity almost three times higher than that of the standard gold chip (Figure 4). Unfortunately, at the same time, it entails a narrowing of the analytically useful range. The linear response range for the biosensor with the bimetallic chip is between LOQ (0.01 ng/mL) and 1 ng/mL, while for the biosensor with a pure gold chip it is between 0.01 and 2 ng/mL. Taking into consideration the fact that levels of VEGF-R2 in blood plasma range from 0.6 to 2.6 ng/mL [this paper] and those in blood serum from approx. 6 to 23 ng/mL [4,5], it is clear that dilution of samples may be necessary. This operation is easier when the linearity range is wider. A similar situation was observed in the case of VEGF. The concentrations of this protein in plasma were lower than in serum. The authors of the paper explain this state of affairs in terms of the probability that VEGF released from platelets has a greater share of serum concentrations. To minimize variations in VEGF concentrations, it is recommended to use EDTA glass tubes instead of their plastic counterparts to minimize the risk of platelet activation [29]. Perhaps a similar relationship exists for VEGF-R2.

Precision and recovery were investigated both under model conditions and by the spiking of blood plasma (Table 3 and Table 4). Surprisingly, the results for the spiked samples are much better that those in the model investigations, especially in the case of the biosensor based on the bimetallic chip. Additionally, the results for the biosensor based on the gold chip are better than those for the bimetallic chip. Despite these differences, the recoveries are not worse than 112%, while the precision is better than 11% in all cases.

Special attention was paid to testing the selectivity of the developed biosensors. VEGF-R1 and VEGF-R3, the other members of VEGF-R family, were selected as potential interferents, as well as VEGF-A and NRP-1, which are involved in angiogenesis jointly with VEGF-R2. Tolerance of VEGF-A is especially significant because VEGF-A reacts with VEGF-R2. None of these potential interferents have a significant influence on the results of VEGF-R2 determination even at 1:100 excess. This high selectivity of the two developed biosensors was confirmed in reverse experiments in which VEGF-A in excess, alone or jointly with NRP-1, interacted with the biosensor already containing entrapped VEGF-R2. The lack of any influence is evidence that VEGF-R2, entrapped by monoclonal rabbit antibody specific for human VEGF-R2, had lost the ability to react with VEGF-A. Thus, excellent selectivity was attained for both versions of the biosensor.

Using QCM and SPRi, basic thermodynamic characteristics of the ligand–VEGF-R2 system were determined. The results are summarized in Table 5. As they are mutually comparable, we may assume that QCM and SPRi can be used alternatively for this type of study. The table also includes information about the surfaces of both biosensors: the theoretical amount of ligands bound on the surface (ligand_sites_) and functional ligands (ligand_functional_) capable of capturing VEGF-R2.

Both biosensors were validated by VEGF-R2 determination in 27 real samples and by parallel determination with an ELISA. The results for both biosensors showed very good agreement, and good agreement was also obtained between the results from the two biosensors showing the high equivalence of the two versions of the biosensor. All of the above features of the newly developed biosensors provide evidence that these biosensors may be very good complementary methods to those currently used for quantifying VEGF-R2 in plasma.

## 4. Materials and Methods

### 4.1. Reagents and Methodology

The function of the VEGF-R2 capture ligand (recombinant human VEGF-R2, Abcam, UK) from the probe was performed by a monoclonal rabbit antibody specific for human VEGF-R2 (Abcam, Cambridge, UK). Recombinant human VEGF-R1, VEGF-R3 and NRP-1 (neuropilin-1) were purchased from Abcam (Cambridge, UK). Commercial ELISA (Abcam, Cambridge, UK) was used as a comparative method. A 99.98% ethyl alcohol (POCh, Gliwice, Poland), EDC, NHS, cysteamine, glycine, BSA and human albumin (all Sigma Aldrich, Steinheim, Germany) were also used during the research. PBS buffer (pH = 7.4) was used to dilute the test samples. The surface of the biosensor was washed with HBS-ES (pH = 7.4) to remove non-specifically bound particles. All solution preparation and rinsing of the biosensor surface were performed with milliQ water.

The QCM investigation was carried out using a quartz crystal microbalance coupled with a PGSTAT 302N potentiostat/galvanostat (Methrom Autolab B.V., Utrecht, The Netherlands). The crystal placed in the measuring cell (3 mL) had a resonance frequency of 6 MHz and an area of 0.361 cm^2^. The gold layer was 100 nm thick. QCM analysis was supported by dedicated NOVA 2.1 software.

SPRi experiments were performed using a stationary device developed by the University of Bialystok and the company AC S.A. The main parts of the SPRi apparatus used were a diode laser emitting a light beam with a length of 635 nm, a fiber optic collimator, a linear polarizer, a glass prism and a chip (in Kretschmann configuration), and a CCD camera as a detector. We used two polarizations: p polarization and s polarization. The p polarization was used to make basic measurements, and the s polarization to measure the background, which was then subtracted. The results were processed using ImageJ software (NIH, version 1.32). The quantity of analyte required for the analysis was only 3 µL.

### 4.2. Biological Material

Plasma samples were taken from patients with diagnosed brain glioma (G1–G4), and blood plasma from smokers was used as a control (K). The samples used for the research came from the Biobank of the Medical University in Bialystok. A total of 27 samples were tested. The study obtained the consent of the relevant bioethical committee (license APK.002.171.2021). The tested samples were diluted so that the range of signals received from the detector lay within the range of the calibration curves. All samples were diluted twice for use with the biosensor with a gold layer, and four times for use with the biosensor with a bimetallic layer.

### 4.3. Statistical Analysis

Statistical analysis was performed using Statistica 13.3 software (TIBCO Software Inc., Palo Alto, CA, USA).

## Figures and Tables

**Table 1:** Types of immobilizations on the biosensor surface.

Type of Immobilization	Strategy Description	Advantages	Disadvantages	Ref.
Immobilization via binding proteins	A layer of proteins is formed on the surface of the biosensor that is specific to the respective regions of the antibodies.	1. Increased sensitivity compared to random immobilization. 2. No need to modify the surface with an antibody. 3. Possibility of multiple regeneration of the biosensor (if cross-linking is used).	1. The use of cross-linking may reduce sensitivity, and without it, regeneration is not possible, making the biosensor disposable. 2. Possibility to use this type of immobilization only for some classes of antibodies.	[9]
Immobilization by antibody fragments	Disruption of disulfide bridges in the antibody and immobilization with sulfhydryl groups	1. Increase in sensitivity compared to random immobilization. 2. The possibility of regulating the affinity of the antibody to the antigen (with the use of recombinant Fab’). 3. The possibility of multiple regeneration.	1. Dense surface packing can cause steric hindrance. 2. Potential loss of antibody biological activity due to chemical reduction. 3. Low stability of antibody fragments. 4. Possible denaturation in case of direct contact of the antibody with the gold surface of the biosensor.	
Immobilization by an oxidized oligosaccharide moiety	Oxidation of the oligosaccharide moiety followed by conjugation to molecules with amino or hydrazine groups	1. Increase in sensitivity compared to random immobilization. 2. No need to modify amino acids. 3. The possibility of multiple regeneration.	1. High impact of changes in analysis conditions. 2. Possible damage to the antibody structure during oxidation.	
Random immobilization and physical adsorption	Formation of a covalent bond by amine coupling	1. Good sensitivity provided the appropriate spatial arrangement of the antibody molecules. 2. The possibility of multiple regeneration.	1. Lower sensitivity for random immobilization compared to targeted immobilization methods. 2. In the case of physical adsorption–denaturation, low stability and random spatial orientation of proteins.	

**Table 2:** Parameters used to model the SPR curves. ε’—real part of the permittivity, ε”—imaginary part of the permittivity.

Gold Chip				Bimetallic Chip			
***prism***		***triangular***		*prism*		*triangular*	
***prism angle***		***60^o^***		*prism angle*		*60^o^*	
**Layer**	**Thickness [nm]**	**ε’**	**ε”**	**Layer**	**Thickness [nm]**	**ε’**	**ε”**
**BK-7**	0	2.29	0	**BK-7**	0	2.29	0
**chromium**	1	−6.3	30	**chromium**	2	−6.3	30
**gold**	46	−12.45	1.3	**silver**	40	−16	0.6
**cysteamine**	1	1.5	0	**gold**	6	−12.45	1.3
**ligand**	1.8	1.55	0	**cysteamine**	1	2.5	0
**VEGF-R2**	2.2	1.57	0	**ligand**	1.2	3	0
				**VEGF-R2**	1.33	4	0

**Table 3:** Validation parameters of the developed methods.

Parameter	Gold Chip			Bimetallic Chip		
	C_RM_ [ng/mL]			C_RM_ [ng/mL]		
	0.030	1.000	2.000	0.030	0.050	1.000
C_quant_ [ng/mL]	0.030	1.030	2.001	0.031	0.056	1.001
SD [ng/mL]	0.002	0.041	0.006	0.001	0.006	0.103
REC [%]	100.00	103.00	100.05	103.33	112.00	100.10
CV [%]	6.60	4.00	0.30	3.23	10.70	10.30
**Analytical limits**						
LOB [ng/mL]	0.005			0.005		
LOD [ng/mL]	0.010			0.010		
LOQ [ng/mL]	0.030			0.030		

**Table 4:** Recovery by the method of standard addition.

Gold Chip						
C_control_ [ng/mL]	C_add_ [ng/mL]	Measurement No	C_theor_ [ng/mL]	C_quant_ [ng/mL]	SD [ng/mL]	REC [%]
1.74 ± 0.05	5.22	1	6.96	7.07	0.09	102.11
		2		7.00	0.10	100.77
		3		6.91	0.08	99.04
		4		7.07	0.09	102.11
		5		7.09	0.06	102.49
Bimetallic chip						
C_control_ [ng/mL]	C_add_ [ng/mL]	Measurement No	C_theor_ [ng/mL]	C_quant_ [ng/mL]	SD [ng/mL]	REC [%]
1.14 ± 0.06	3.42	1	4.56	4.69	0.07	103.80
		2		4.54	0.10	99.56
		3		4.57	0.09	99.42
		4		4.65	0.07	102.63
		5		4.63	0.05	102.05

**Table 5:** Summary of the characteristics of the tested biological system.

Thermodynamics Parameters		
	QCM	SPRi
K_D_	2.10 × 10^−12^ M	2.05 × 10^−12^ M
K_A_	4.73 × 10^11^ 1/M	4.00 × 10^11^ 1/M
**Surface parameters**		
	**Gold chip**	**Bimetallic chip**
ligand_sites_	13.51 pmol/mm^2^ (2.04 × 10^3^ ng/mm^2^)	21.55 pmol/mm^2^ (3.25 × 10^3^ ng/mm^2^)
ligand_functional_	99.99%	99.82 %

## Data Availability

Not applicable.

## Sample Availability

Samples of the compounds are available from the authors.

## Appendix B. Robustness of Analytical Methods

To determine the influence of changes in the analytical procedure on the stability of the results obtained, a robustness test of the analytical method was carried out. Table A1 shows the changes in the analytical procedure and their effect on the quantification results. Each measurement was carried out in triplicate.

**Table A1:** Effect of changes in the analytical procedure on the value of the quantification result.

Change in the Analytical Procedure	Description of the Change in the Analytical Procedure	VEGF-R2			
		Gold Chip		Bimetallic Chip	
		X_C_ [ng/mL]	SD [ng/mL]	X_C_ [ng/mL]	SD [ng/mL]
-	sample prepared on the day of analysis (nominal value)	1.78	0.03	1.22	0.08
T↑	sample heated in an incubator for 2 h (40 °C)	1.08	0.05	0.89	0.02
T↓	sample chilled in the refrigerator for 2 h (4 °C)	1.75	0.07	1.29	0.04
pH < 7.40	pH = 4.99	1.26	0.04	0.94	0.07
pH > 7.40	pH = 9.50	1.18	0.06	1.08	0.04
shot and gun	sample prepared immediately before the analysis	1.83	0.05	1.19	0.03
time of measurement (interaction of sample with biosensor)	change in sample measurement time (sample prepared on the day of analysis)				
	30 s	0.29	0.04	0.36	0.02
	2 min	0.95	0.06	0.84	0.05
	5 min	1.69	0.03	1.12	0.02
	8 min	1.77	0.05	1.25	0.04
	10 min	1.68	0.04	1.19	0.03

X_C_—mean concentration in the sample. SD—standard deviation.

The above experiments show that the changes in the analysis time, for both the gold and the bimetallic chip, had the greatest impact on the stability of the obtained results. Concentration values begin to stabilize after approximately 5 min. The optimal time for this analysis is 8–10 min. In addition, the temperature increase over 2 h has a major influence. Changes in pH have a slightly smaller influence (the optimum pH for the analysis is 7.40). It was not observed that the cooling of the sample (to 4 °C) or its preparation immediately before the analysis had a real impact on the results obtained.

It was also investigated how many times it is possible to regenerate biosensors with a glycine–HCl solution at pH = 2.50 and reuse them for quantification without a significant decrease in their precision and accuracy. The regeneration of the biosensor surface was carried out by applying 3 µL of the glycine–HCl solution several times to the surface of the biosensor. The entire process took no more than 45 s. Regeneration with a glycine–HCl solution allowed the removal of ligand-bound analyte molecules and the reuse of the same biosensor plate. The same biological sample was prepared immediately before the analyses were used for the research. The test results are summarized in Table A2.

**Table A2:** Investigation of the influence of the number of regeneration cycles on quantification.

Regeneration Cycle Number	VEGF-R2			
	Gold Chip		Bimetallic Chip	
	X_C_	SD	X_C_	SD
0	1.76	0.04	1.25	0.02
1	1.73	0.03	1.19	0.06
2	1.69	0.06	1.24	0.04
3	1.75	0.04	1.22	0.03
4	1.78	0.07	1.20	0.07
5	1.71	0.03	1.12	0.03
6	1.34	0.05	1.04	0.07
7	1.32	0.05	0.78	0.03
8	1.09	0.09	0.45	0.07

X_C_—mean concentration in the sample. SD—standard deviation.

There was a noticeable decrease in the determined concentration compared with the original result: for the gold chip after the 6th cycle of biosensor surface regeneration (a difference of 23.86%), and for the bimetallic chip after the 5th regeneration cycle (a difference of 10.40%). Therefore, since each of the biosensors has nine active sites, it is possible to make a maximum of five series of quantitative determinations (45 samples) using the gold chip and four series (36 samples) using a bimetallic chip, where quantification is carried out using the same biosensor plate.

## References

[1] Modi S.J., Kulkarni V.M. (2019). Vascular Endothelial Growth Factor Receptor (VEGFR-2)/KDR Inhibitors: Medicinal Chemistry Perspective. Med. Drug Discov..

[2] Fuh G., Li B., Crowley C., Cunningham B., Wells J.A. (1998). Requirements for binding and signaling of the kinase domain receptor for vascular endothelial growth factor. J. Biol. Chem..

[3] Pezzilli R., Fabbri D., Corsi M.M., Imbrogno A., Barassi A., Morselli-Labate A.M., Dogliotti G., Casadei R., Corinaldesi R., D’Eril G.M. (2011). Plasma concentrations of angiogenetic factors and angiogenetic inhibitors in patients with ductal pancreatic neoplasms. A pilot study. Clin. Chem. Lab. Med..

[4] Kotowicz B., Fuksiewicz M., Jonska-Gmyrek J., Berezowska A., Radziszewski J., Bidzinski M., Kowalska M. (2017). Clinical significance of pretreatment serum levels of VEGF and its receptors, IL- 8, and their prognostic value in type I and II endometrial cancer patients. PLoS ONE.

[5] Meena R., Nangia A., Sharma S., Chandra J. (2021). Serum Levels of Vascular Endothelial Growth Factor and Its Receptor in Newly Diagnosed Paediatric Acute Lymphoblastic Leukemia. Indian J. Hematol. Blood Transfus..

[6] Skirnisdottir I., Akerud H., Seidal T. (2018). Clinical significance of growth factor receptor EGFR and angiogenesis regulator VEGF-R2 in patients with ovarian cancer at FIGO stages I-II. Int. J. Oncol..

[7] Skirnisdottir I., Seidal T., Akerud H. (2016). The relationship of the angiogenesis regulators VEGF-A, VEGF-R1 and VEGF-R2 to p53 status and prognostic factors in epithelial ovarian carcinoma in FIGO-stages I-II. Int. J. Oncol..

[8] Wei T., Tu W., Zhao B., Lan Y., Bao J., Dai Z. (2014). Electrochemical monitoring of an important biomarker and target protein: VEGFR2 in cell lysates. Sci. Rep..

[9] Makaraviciute A., Ramanaviciene A. (2013). Site-directed antibody immobilization techniques for immunosensors. Biosens. Bioelectron..

[10] Ladd J., Taylor A.D., Piliarik M., Homola J., Jiang S. (2009). Label-free detection of cancer biomarker candidates using surface plasmon resonance imaging. Anal. Bioanal. Chem..

[11] Li Y., Hye J.L., Corn R.M. (2007). Detection of protein biomarkers using RNA aptamer microarrays and enzymatically amplified surface plasmon resonance imaging. Anal. Chem..

[12] Shabani A., Tabrizian M. (2013). Design of a universal biointerface for sensitive, selective, and multiplex detection of biomarkers using surface plasmon resonance imaging. Analyst.

[13] Zhu L., Wang K., Cui J., Liu H., Bu X., Ma H., Wang W., Gong H., Lausted C., Hood L. (2014). Label-free quantitative detection of tumor-derived exosomes through surface plasmon resonance imaging. Anal. Chem..

[14] Suwansa-ard S., Kanatharana P., Asawatreratanakul P., Wongkittisuksa B., Limsakul C., Thavarungkul P. (2009). Comparison of surface plasmon resonance and capacitive immunosensors for cancer antigen 125 detection in human serum samples. Biosens. Bioelectron..

[15] Szymanska B., Lukaszewski Z., Zelazowska-Rutkowska B., Hermanowicz-Szamatowicz K., Gorodkiewicz E. (2021). An spri biosensor for determination of the ovarian cancer marker he4 in human plasma. Sensors.

[16] Altintas Z., Uludag Y., Gurbuz Y., Tothill I.E. (2011). Surface plasmon resonance based immunosensor for the detection of the cancer biomarker carcinoembryonic antigen. Talanta.

[17] Wong C.L., Loke S.Y., Lim H.Q., Balasundaram G., Chan P., Chong B.K., Tan E.Y., Lee A.S.G., Olivo M. (2021). Circulating microRNA breast cancer biomarker detection in patient sera with surface plasmon resonance imaging biosensor. J. Biophotonics.

[18] Sina A.A.I., Vaidyanathan R., Wuethrich A., Carrascosa L.G., Trau M. (2019). Label-free detection of exosomes using a surface plasmon resonance biosensor. Anal. Bioanal. Chem..

[19] Lu X., Tao L., Song D., Li Y., Gao F. (2018). Bimetallic Pd@Au nanorods based ultrasensitive acetylcholinesterase biosensor for determination of organophosphate pesticides. Sens. Actuators B Chem..

[20] Xia L., Yin S., Gao H., Deng Q., Du C. (2011). Sensitivity Enhancement for Surface Plasmon Resonance Imaging Biosensor by Utilizing Gold-Silver Bimetallic Film Configuration. Plasmonics.

[21] Liu L., Wang M., Jiao L., Wu T., Xia F., Liu M., Kong W., Dong L., Yun M. (2019). Sensitivity enhancement of a graphene–barium titanate-based surface plasmon resonance biosensor with an Ag–Au bimetallic structure in the visible region. J. Opt. Soc. Am. B.

[22] Falkowski P., Mrozek P., Lukaszewski Z., Oldak L., Gorodkiewicz E. (2021). An immunosensor for the determination of cathepsin s in blood plasma by array spri—A comparison of analytical properties of silver–gold and pure gold chips. Biosensors.

[23] Mrozek P., Gorodkiewicz E., Falkowski P., Hościło B. (2021). Sensitivity analysis of single-and bimetallic surface plasmon resonance biosensors. Sensors.

[24] Karlsson R., Michaelsson A., Mattsson L. (1991). Kinetic analysis of monoclonal antibody-antigen interactions with a new biosensor based analytical system. J. Immunol. Methods.

[25] Stenberg E., Persson B., Roos H., Urbaniczky C. (1991). Quantitative determination of surface concentration of protein with surface plasmon resonance using radiolabeled proteins. J. Colloid Interface Sci..

[26] Marquart A., Kuncova-Kallio J., Albers M., Bombera R., Stahlberg R.K. (2019). Handbook of MP-SPR.

[27] Naik G., Kim J., Kinsey N., Boltasseva A. (2014). Alternative plasmonic materials. Handbook of Surface Science.

[28] Ma L., Gu K., Zhang C.H., Chen X.T., Jiang Y., Melcher K., Zhang J., Wang M., Xu H.E. (2016). Generation and characterization of a human nanobody against VEGFR-2. Acta Pharmacol. Sin..

[29] McIlhenny C., George W.D., Doughty J.C. (2002). A comparison of serum and plasma levels of vascular endothelial growth factor during the menstrual cycle in healthy female volunteers. Br. J. Cancer.

